# Supplementary material for: Reproducibility-driven discovery and systematic benchmarking reveal a robust cerebrospinal fluid proteomic signature in Alzheimer’s disease
Source: medRxiv. 2026 Apr 28:2026.04.26.26351766. Preprint. [Version 1] doi: 10.64898/2026.04.26.26351766 (PMC13142593; doi:10.64898/2026.04.26.26351766)
Supplement: Supplement 1 [file media-1.pdf]

Supplemental Information

Reproducibility-driven discovery and systematic benchmarking reveal a robust cerebrospinal fluid proteomic signature in Alzheimer's disease

María Fernanda Zambrano-Astorga and Aldo Moreno-Ulloa, for the Alzheimer's Disease Neuroimaging Initiative**

[1]Life Sciences Graduate Student Program, Center for Scientific Research and Higher Education at Ensenada, Baja California (CICESE), Carretera Ensenada-Tijuana No. 3918, Zona Playitas, C.P. 22860, Ensenada, Baja California, Mexico.

[2]Biomedical Innovation Department, Center for Scientific Research and Higher Education at Ensenada, Baja California (CICESE), Carretera Ensenada-Tijuana No. 3918, Zona Playitas, C.P. 22860, Ensenada, Baja California, Mexico.

***Corresponding author:** amoreno@cicese.mx

**Data used in preparation of this article were obtained from the Alzheimer's Disease Neuroimaging Initiative (ADNI) database (adni.loni.usc.edu). As such, the investigators within the ADNI contributed to the design and implementation of ADNI and/or provided data but did not participate in analysis or writing of this report. A complete listing of ADNI investigators can be found at: http://adni.loni.usc.edu/wp-content/uploads/how_to_apply/ADNI_Acknowledgement_List.pdf

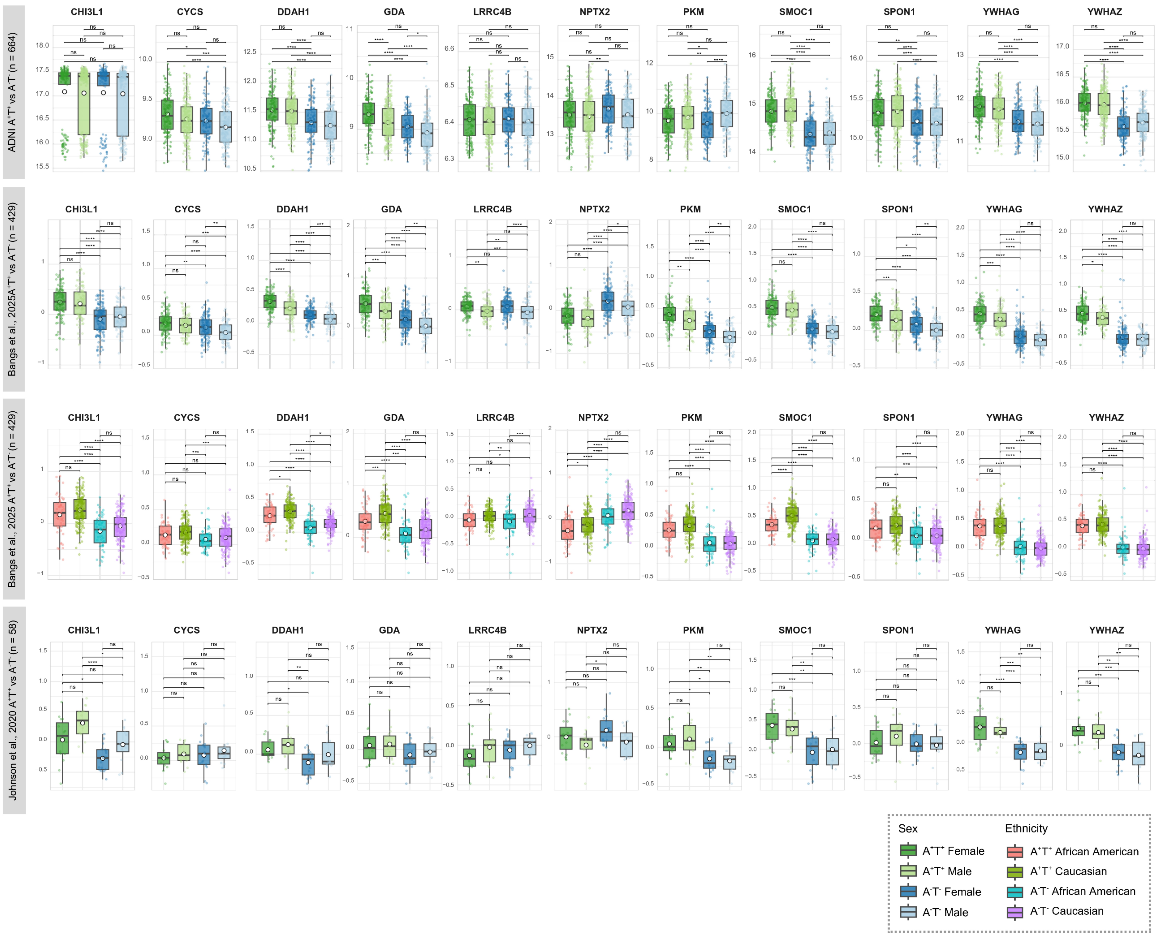

Figure S1. Differential abundance of PPAV11 proteins between biological Alzheimer's disease (AD) and control subjects stratified by sex and ethnicity. Log_2 abundance of proteins in ADNI (by sex), Bangs et al.[1] (by sex and ethnicity) and Johnson et al.[2] (by sex) comparing A^+T^+ and A^-T^- subjects. Box plots represent the median and interquartile range, while mean estimates are by white dots. Significance was assessed by one-way ANOVA followed by Tukey's post hoc test, *p-adjusted < 0.05, **p-adjusted < 0.01, ***p-adjusted < 0.001, ****p-adjusted < 0.0001, ns = non-significant. Abbreviations: ADNI, Alzheimer's Disease Neuroimaging Initiative; A, Amyloid; T, Tau.

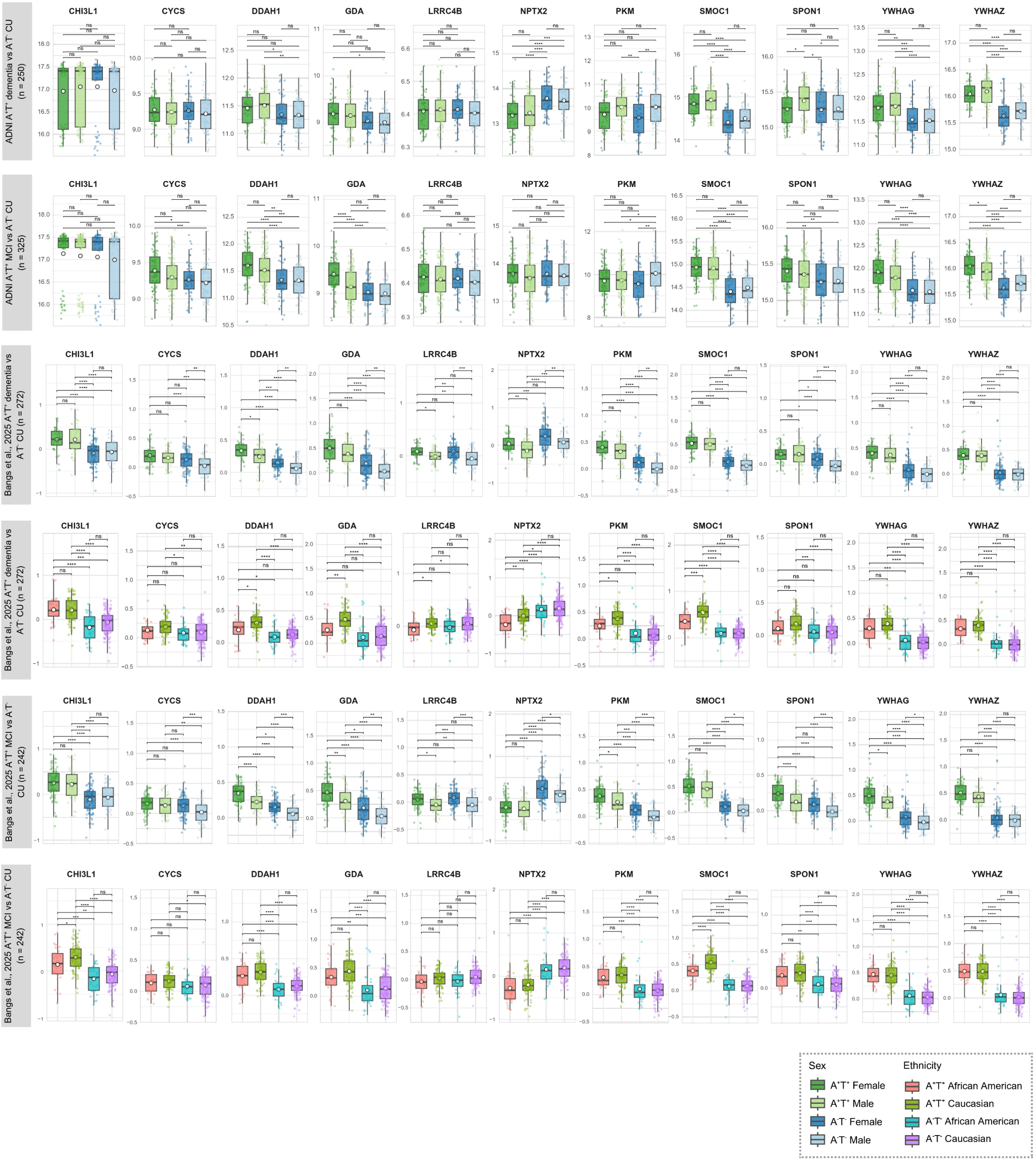

Figure S2. Differential abundance of PPAV11 proteins between biological and clinical Alzheimer's disease (AD) and control subjects stratified by sex and ethnicity. Log_2 abundance of proteins in ADNI (by sex), Bangs et al.[1] (by sex and ethnicity) and Johnson et al.[2] (by sex) comparing A^+T^+ cognitively impaired (MCI or dementia) and A^-T^- CU subjects. Box plots represent the median and interquartile range, while mean estimates are by white dots. Significance was assessed by one-way ANOVA followed by Tukey's post hoc test, *p-adjusted < 0.05, **p-adjusted < 0.01, ***p-adjusted < 0.001, ****p-adjusted < 0.0001, ns = non-significant. Abbreviations: ADNI, Alzheimer's Disease Neuroimaging Initiative; A, Amyloid; T, tau; CU, cognitively unimpaired; MCI, mild cognitively impaired.

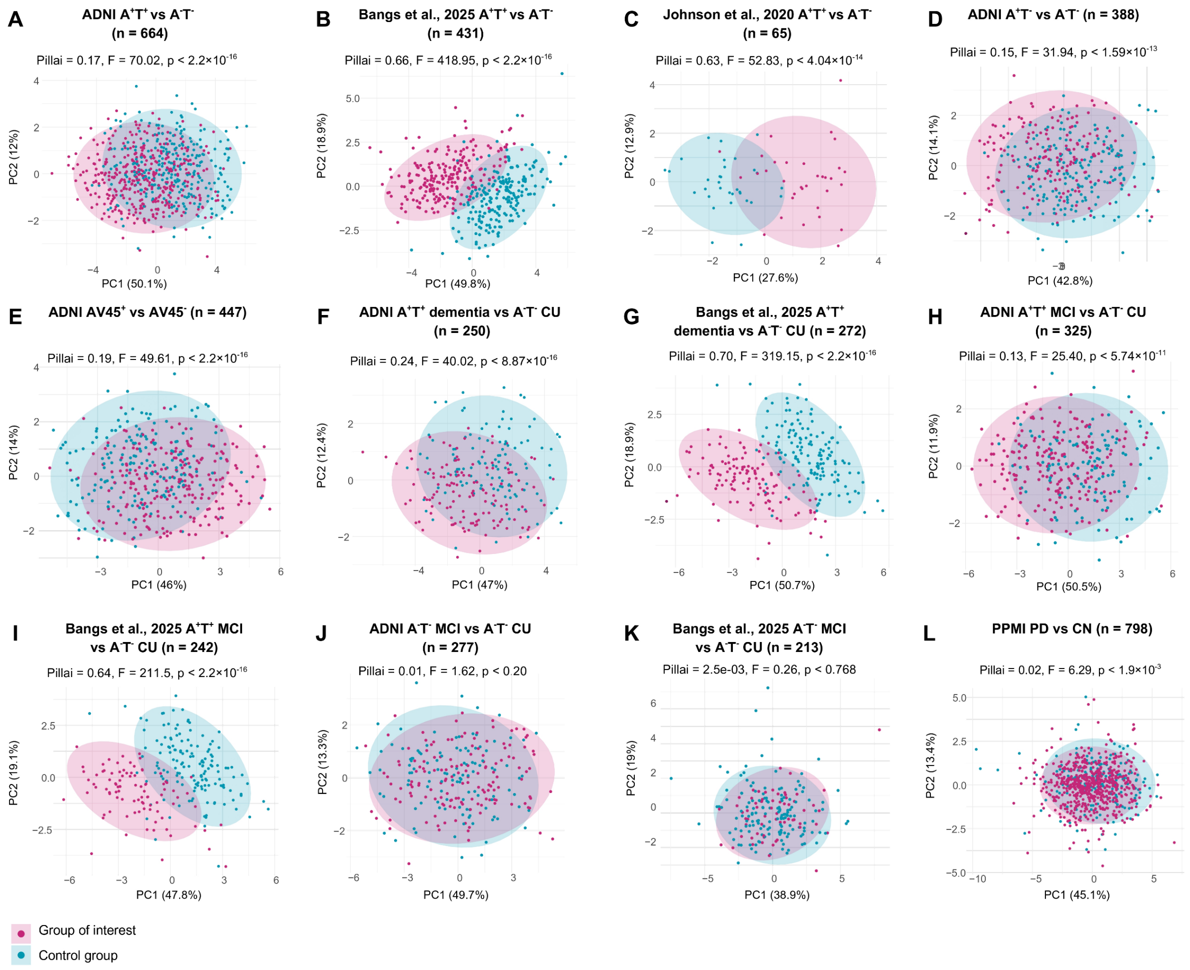

Figure S3. Differentiation between biological or clinical Alzheimer's disease (AD) and control groups by PPAV11 protein abundances using principal component analysis (PCA). Subjects stratified by (A-E) biological diagnosis (A^+T^+ vs A^-T^-, A^+T^- vs A^-T^-, and $AV45^+$ vs $AV45^-$); and by (F-I) biological diagnosis and clinical status (A^+T^+ MCI vs A^-T^- CU, and A^+T^+ dementia vs A^-T^- CU). Selectivity of PPAV11 evaluated by comparing (J-K) A^-T^- MCI vs A^-T^- CU subjects, and (L) PD vs control subjects. Significance was assessed by Pillai's trace (MANOVA). Ellipses represent 95% confidence intervals. Abbreviations: ADNI, Alzheimer's Disease Neuroimaging Initiative; A, Amyloid β_{42}; T, Tau; AV45, Florbetapir F18 positron emission tomography; MCI, mild cognitively impaired; CU, cognitively unimpaired; PPMI, Parkinson's Progression Markers Initiative; PD, Parkinson's disease; PET, positron emission tomography; CSF, cerebrospinal fluid; MANOVA, multivariate analysis of variance.

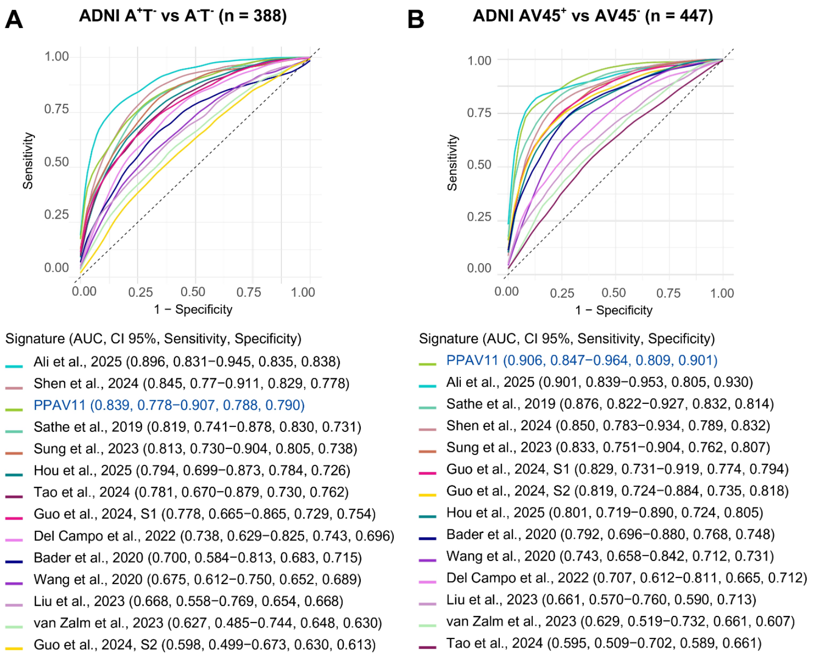

Figure S4. Receiver operating characteristics (ROC) curve analysis to distinguish between subjects with abnormal and normal brain and CSF levels of Aβ. Subjects stratified by abnormal and normal in the ADNI cohort by (A) $A\beta_{42}$ CSF levels, and (B) 18F-AV45 brain levels. Comparative analysis was performed against Ali et al.[3], Bader et al.[4], Del Campo et al.[5], Guo et al.[6], Hou et al.[7], Liu et al.[8], Sathe et al.[9], Shen et al.[10], Sung et al.[11], Tao et al.[12], van Zalm et al.[13], and Wang et al.[14]. Sensitivity and specificity were determined by Youden's index. Abbreviations: ADNI, Alzheimer's Disease Neuroimaging Initiative; A, Amyloid β_{42}; T, Tau; AV45, Florbetapir F18 positron emission tomography; AUC, area under the curve; CI, confidence intervals; CSF, cerebrospinal fluid.

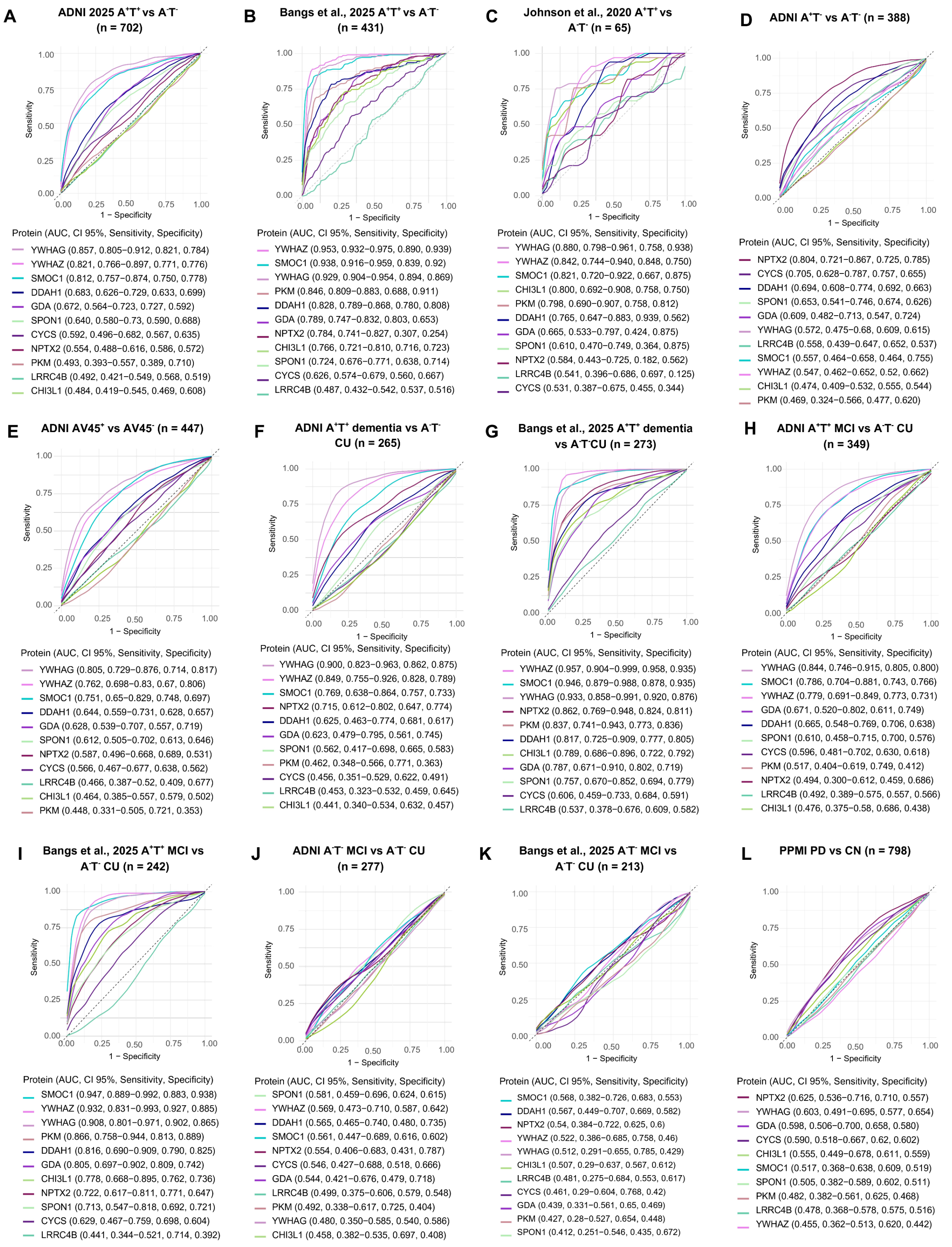

Figure S5. Validation of individual proteins from PPAV11 signature for biological and clinical Alzheimer's disease (AD) diagnosis. Receiver operating characteristics (ROC) curve analyses

comparing the utility of single proteins to distinguish between subjects stratified by (A-E) biological diagnosis (A^+T^+ vs A^-T^-, A^+T^- vs A^-T^-, and $AV45^+$ vs $AV45^-$); and by (F-I) biological diagnosis and clinical status (A^+T^+ MCI vs A^-T^- CU, and A^+T^+ dementia vs A^-T^- CU). Selectivity of PPAV11 evaluated by comparing (J-K) A^-T^- MCI vs A^-T^- CU subjects, and (L) PD vs control subjects. Sensitivity and specificity were determined by Youden's index. Abbreviations: ADNI, Alzheimer's Disease Neuroimaging Initiative; A, Amyloid β_{42}; T, Tau; AV45, Florbetapir F18 positron emission tomography; CU, cognitively unimpaired; MCI, mild cognitively impaired; PPMI, Parkinson's Progression Markers Initiative; PD, Parkinson's Disease; CN, control; AUC, area under the curve; CI, confidence intervals.

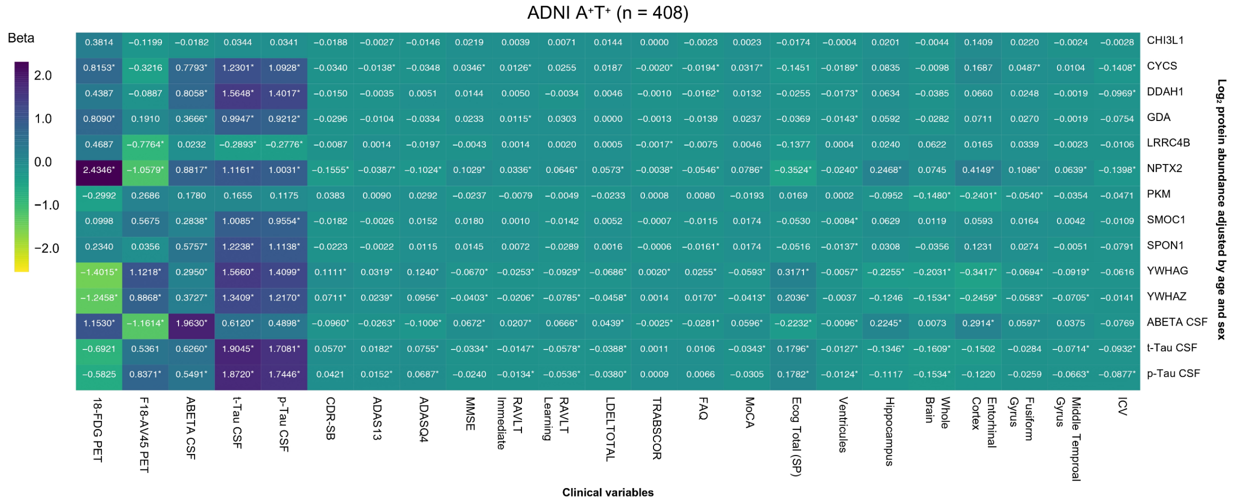

Figure S6. Correlation analysis of PPAV11 proteins with clinical variables in the ADNI cohort. Heatmap shows correlations of individual PPAV11 proteins and core AD biomarkers with cognitive tests, brain volume measurements, and biomarkers. Beta coefficient values were derived from LIMMA analysis and represent the association of change in protein abundance (normalized by z-score) per unit of each clinical variable. Models were adjusted for age and sex. MRI volume metrics are expressed in cm^2. Significance (p-adjusted < 0.05, Benjamini-Hochberg correction) is highlighted by an asterisk. Abbreviations: ADNI, Alzheimer's Disease Neuroimaging Initiative; 18-FDG PET, ^{18}F-fluorodeoxyglucose positron emission tomography; F18-AV45 PET, Florbetapir F18 positron emission tomography; t-Tau, total Tau; p-Tau, phosphorylated Tau; CDR-SB, Clinical Dementia Rating–Sum of Boxes; ADAS, Alzheimer's Disease Assessment Scale; MMSE, Mini Mental State Examination; RAVLT, Rey Auditory Verbal Learning Test; LDEDLTOTAL, logical memory delayed recall total; TRABSCOR, Trail Making Test; FAQ, Functional Activities Questionnaire; MoCA, Montreal Cognitive Assessment; ECOG (SP), Everyday Cognition, spouse report; ICV, intracerebroventricular.

References

1. Bangs, M.C., Gadhavi, J., Carter, E.K., Ping, L., Duong, D.M., Dammer, E.B., Wu, F., Shantaraman, A., Fox, E.J., Johnson, E.C.B., et al. (2025). Proteomic Subtyping of Alzheimer's Disease CSF links Blood-Brain Barrier Dysfunction to Reduced levels of Tau and Synaptic Biomarkers. bioRxiv. 10.1101/2025.03.14.643332.
2. Johnson, E.C.B., Dammer, E.B., Duong, D.M., Ping, L., Zhou, M., Yin, L., Higginbotham, L.A., Guajardo, A., White, B., Troncoso, J.C., et al. (2020). Large-scale proteomic analysis of Alzheimer's disease brain and cerebrospinal fluid reveals early changes in energy metabolism associated with microglia and astrocyte activation. Nat Med *26*, 769-780. 10.1038/s41591-020-0815-6.
3. Ali, M., Timsina, J., Western, D., Liu, M., Beric, A., Budde, J., Do, A., Heo, G., Wang, L., Gentsch, J., et al. (2025). Multi-cohort cerebrospinal fluid proteomics identifies robust molecular signatures across the Alzheimer disease continuum. Neuron *113*, 1363-1379 e1369. 10.1016/j.neuron.2025.02.014.
4. Bader, J.M., Geyer, P.E., Muller, J.B., Strauss, M.T., Koch, M., Leypoldt, F., Koertvelyessy, P., Bittner, D., Schipke, C.G., Incesoy, E.I., et al. (2020). Proteome profiling in cerebrospinal fluid reveals novel biomarkers of Alzheimer's disease. Mol Syst Biol *16*, e9356. 10.15252/msb.20199356.
5. Del Campo, M., Peeters, C.F.W., Johnson, E.C.B., Vermunt, L., Hok, A.H.Y.S., van Nee, M., Chen-Plotkin, A., Irwin, D.J., Hu, W.T., Lah, J.J., et al. (2022). CSF proteome profiling across the Alzheimer's disease spectrum reflects the multifactorial nature of the disease and identifies specific biomarker panels. Nat Aging *2*, 1040-1053. 10.1038/s43587-022-00300-1.
6. Guo, Y., Chen, S.D., You, J., Huang, S.Y., Chen, Y.L., Zhang, Y., Wang, L.B., He, X.Y., Deng, Y.T., Zhang, Y.R., et al. (2024). Multiplex cerebrospinal fluid proteomics identifies biomarkers for diagnosis and prediction of Alzheimer's disease. Nat Hum Behav *8*, 2047-2066. 10.1038/s41562-024-01924-6.
7. Hou, X., Qiu, Y., Li, H., Yan, Y., Zhao, D., Ji, S., Ni, J., Zhang, J., Liu, K., Qing, H., and Quan, Z. (2025). Machine-learning based strategy identifies a robust protein biomarker panel for Alzheimer's disease in cerebrospinal fluid. Alzheimers Res Ther *17*, 147. 10.1186/s13195-025-01789-5.
8. Liu, P., Li, L., He, F., Meng, F., Liu, X., Su, Y., Su, X., Luo, B., and Peng, G. (2023). Identification of Candidate Biomarkers of Alzheimer's Disease via Multiplex Cerebrospinal Fluid and Serum Proteomics. Int J Mol Sci *24*. 10.3390/ijms241814225.
9. Sathe, G., Na, C.H., Renuse, S., Madugundu, A.K., Albert, M., Moghekar, A., and Pandey, A. (2019). Quantitative Proteomic Profiling of Cerebrospinal Fluid to Identify Candidate Biomarkers for Alzheimer's Disease. Proteomics Clin Appl *13*, e1800105. 10.1002/prca.201800105.
10. Shen, Y., Timsina, J., Heo, G., Beric, A., Ali, M., Wang, C., Yang, C., Wang, Y., Western, D., Liu, M., et al. (2024). CSF proteomics identifies early changes in autosomal dominant Alzheimer's disease. Cell *187*, 6309-6326 e6315. 10.1016/j.cell.2024.08.049.
11. Sung, Y.J., Yang, C., Norton, J., Johnson, M., Fagan, A., Bateman, R.J., Perrin, R.J., Morris, J.C., Farlow, M.R., Chhatwal, J.P., et al. (2023). Proteomics of brain, CSF, and plasma identifies molecular signatures for distinguishing sporadic and genetic Alzheimer's disease. Sci Transl Med *15*, eabq5923. 10.1126/scitranslmed.abq5923.
12. Tao, Q.Q., Cai, X., Xue, Y.Y., Ge, W., Yue, L., Li, X.Y., Lin, R.R., Peng, G.P., Jiang, W., Li, S., et al. (2024). Alzheimer's disease early diagnostic and staging biomarkers revealed by large-scale cerebrospinal fluid and serum proteomic profiling. Innovation (Camb) *5*, 100544. 10.1016/j.xinn.2023.100544.
13. van Zalm, P.W., Ahmed, S., Fatou, B., Schreiber, R., Barnaby, O., Boxer, A., Zetterberg, H., Steen, J.A., and Steen, H. (2023). Meta-analysis of published cerebrospinal fluid proteomics data identifies and validates metabolic enzyme panel as Alzheimer's disease biomarkers. Cell Rep Med *4*, 101005. 10.1016/j.xcrm.2023.101005.
14. Wang, H., Dey, K.K., Chen, P.C., Li, Y., Niu, M., Cho, J.H., Wang, X., Bai, B., Jiao, Y., Chepyala, S.R., et al. (2020). Integrated analysis of ultra-deep proteomes in cortex, cerebrospinal fluid and serum reveals a mitochondrial signature in Alzheimer's disease. Mol Neurodegener *15*, 43. 10.1186/s13024-020-00384-6.
